# Harvesting AAV by tangential flow filtration using reverse asymmetric membranes

**DOI:** 10.1002/btpr.70059

**Published:** 2025-08-04

**Authors:** Xiaolei Hao, Ronny Horax, Xianghong Qian, April Wheeler, Hironobu Shirataki, S. Ranil Wickramasinghe

**Affiliations:** ^1^ Department of Biomedical Engineering University of Arkansas Fayetteville Arkansas USA; ^2^ Ralph E. Martin Department of Chemical Engineering University of Arkansas Fayetteville Arkansas USA; ^3^ Asahi Kasei Bioprocess America, Inc., Senior Product Manager, Microfiltration Technologies Glenview Illinois USA; ^4^ Scientific Affairs Group, Bioprocess Division Asahi Kasei Life Science Corporation Tokyo Japan

**Keywords:** adeno associated virus, asymmetric membrane, bioreactor clarification, critical flux, tangential flow filtration, transmembrane pressure, virus recovery

## Abstract

Efficient bioreactor clarification for harvesting virus particles is often challenging. Tangential flow filtration is attractive as it can be easily adapted for batch and perfusion operations. Here the feasibility of using reverse asymmetric hollow fiber membranes, where the more open support structure faces the feed stream, has been investigated for harvesting adeno associated virus serotype 2. The open support structure of these membranes stabilizes a secondary membrane consisting of rejected particulate matter. It is essential that the stabilized secondary membrane remains highly permeable. Flux stepping experiments were conducted in total recycle mode in order to determine the critical flux. The critical flux is the maximum stable flux. Higher fluxes lead to a rapid increase in transmembrane pressure under constant flux operation. The critical flux is shown to increase with increasing wall shear rate (feed flow rate). The reduction in turbidity of the permeate relative to the feed decreases with increasing wall shear rate. Harvesting adeno associated virus was conducted at a wall shear rate of 2000 s^−1^. The permeate flux was set at 15 Lm^−2^ h^−1^. The feed was concentrated till the transmembrane pressure reached 3.5 kPa. Diafiltration then commenced using 3 diavolumes. While commencing diafiltration with a smaller feed volume will reduce diluent usage and dilution of the product, it is essential that the transmembrane pressure is not too high to create a compacted low permeability secondary membrane. Here the transmembrane pressure was almost constant at 3.5 kPa during diafiltration. Virus recovery was 94%.

## 1 INTRODUCTION

The manufacture of biopharmaceutical products can be divided into upstream operations that focus on cell culture in order to produce the desired therapeutic, and downstream purification operations that are dedicated to the recovery and purification of the therapeutic product. Today, the development of efficient “mid‐stream” operations for bioreactor harvesting or clarification prior to the first downstream purification product capture operation is often challenging. Cell densities above 1 × 10^8^ cells mL^−1^ are obtained due to improvements in upstream cell culture operations.^1^ Standard unit operations include centrifugation, expanded bed chromatography, depth filtration, and tangential flow filtration (TFF). However, the high cell densities achieved today result in the use of more than one unit operation for bioreactor clarification. In the case of protein‐based therapeutics, a particle‐free product stream is desired which can be fed directly to the first downstream capture operation.

TFF has many advantages over other bioreactor clarification operations.^2^ Unlike centrifugation, if an appropriate pore size membrane is used, e.g., 0.22 μm, 0.45 μm, the particle free permeate can be directly fed to the next capture step. Today there is a great deal of interest in continuous biomanufacturing.^3^, ^4^ For the continuous manufacture of monoclonal antibodies, continuous centrifugation, depth filtration, and TFF have been used. At high cell densities, continuous centrifugation requires frequent desludging that leads to a decrease in product recovery.^5^ Often a secondary clarification operation is required, such as depth filtration.^6^ Similarly, a single unit clarification using depth filtration and TFF is generally not feasible.

Here we focus on TFF for virus harvesting for the production of virus particles for applications in gene therapy and viral vaccines. It is possible to apply TFF for both batch and continuous (perfusion) cell culture processes. Perfusion cell culture operation leads to significant process intensification.^7^ In the case of virus harvesting, the situation is complicated as the desired product is the virus particles. Table 1 summarizes some of the recent literature studies that describe methods for virus harvesting.

**TABLE 1:** Recent research on virus harvesting.

Virus being produced	Type of cell culture operation	Virus harvesting operations	References
Human papillomavirus type‐6 (HPV‐6) L1 virus‐like particles (VLPs), for developing HPV vaccines.	Batch	TFF	Developed a scalable production process for truncated human papillomavirus type‐6 L1 protein using WAVE Bioreactor and hollow fiber membrane.^8^
HIV‐virus like particles (VLPs), for developing HIV vaccines.	Batch	TFF	Use of hollow fiber tangential flow filtration for the recovery and concentration of HIV virus‐like particles produced in insect cells.^9^
Adeno‐associated viruses (AAVs), viral vector for gene therapy.	Mixed: batch and perfusion	For batch: dead‐end, TFDF, and depth filtration For perfusion: ATF and TFDF	AAV process intensification by perfusion bioreaction and integrated clarification.^10^
Lentivirus, viral vector for gene therapy.	Perfusion	TFDF	Process intensification for lentiviral vector manufacturing using TFDF.^11^
Two recombinant vesicular stomatitis virus (rVSV)‐based vectors, for developing vector‐based vaccines	Perfusion	ATF and TFDF	Production of recombinant vesicular stomatitis virus‐based vectors by tangential flow depth filtration.^12^
Defective interfering influenza A virus particles, for interfering with replication of influenza A virus	Perfusion	ATF	Cell culture‐based production of defective interfering influenza A virus particles in perfusion mode using an alternating tangential flow filtration system.^13^
Vaccinia Ankara virus, vaccine against smallpox and mpox	Mixed: batch, semi batch and perfusion	For batch: acoustic settler (AS) and inclined settler (IS) For semi batch and perfusion: AS, IS, and ATF.	Production of Modified Vaccinia Ankara Virus by Intensified Cell Cultures: A Comparison of Platform Technologies for Viral Vector Production.^14^
Influenza A virus, for developing influenza vaccines.	Perfusion	ATF and Acoustic settler	Performance of an acoustic settler versus a hollow fiber–based ATF technology for influenza virus production in perfusion.^15^
rVSV‐NDV, recombinant vesicular stomatitis virus (rVSV)‐based fusogenic oncolytic virus (OV), rVSV‐Newcastle disease virus (NDV), a virus for cancer treatment.	Perfusion	Acoustic settler	Process intensification strategies toward cell culture‐based high‐yield production of a fusogenic oncolytic virus.^16^
Modified Vaccinia virus Ankara (MVA), for treating cancer and challenging pathogens	Mixed: batch and perfusion	For batch: acoustic settler and depth filtration For perfusion: acoustic settler	A high cell density perfusion process for Modified Vaccinia virus Ankara production: Process integration with inline DNA digestion and cost analysis.^17^
A recombinant vesicular stomatitis virus (rVSV), which expresses the spike protein of SARS‐CoV‐2, for developing COVID‐19 vaccines.	Mixed: batch and perfusion	For batch: centrifugation and dead‐end filtration For perfusion: acoustic filter	Development of an Integrated Continuous Manufacturing Process for the rVSV‐Vectored SARS‐CoV‐2 Candidate Vaccine.^18^
Influenza A virus, for developing influenza vaccines.	Perfusion	Inclined settler	Application of an Inclined Settler for Cell Culture‐Based Influenza A Virus Production in Perfusion Mode.^19^
Orf virus (ORFV), a viral vector against infectious diseases and cancer	Batch	Depth filtration	Efficient and scalable clarification of Orf virus from HEK suspension for vaccine development.^20^
A recombinant vesicular stomatitis virus (rVSV)	Batch	Centrifugation, dead‐end, and depth filtration	Purification of recombinant vesicular stomatitis virus‐based HIV vaccine candidate.^21^
Human influenza A virus (H1N1), for developing influenza vaccines.	Batch	A combination of continuous flow centrifugation and prefiltering with Sephadex G‐50.	Downstream processing of Vero cell‐derived human influenza A virus (H1N1) grown in serum‐free medium.^22^
Hepatitis C virus (HCV), for developing HCV vaccines.	Fed‐batch	Centrifugation	High‐Titer Hepatitis C Virus Production in a Scalable Single‐Use High Cell Density Bioreactor.^23^
Adeno‐associated viruses (AAVs), a viral vector for gene therapy.	Batch	Centrifugation	Advanced biomanufacturing and evaluation of adeno‐associated virus.^24^
Oncolytic viruses (OV), for treating solid cancers	Batch	Dead‐end filtration	Streamlining the purification of a clinical‐grade oncolytic virus for therapeutic applications.^25^
Adeno‐associated viruses (AAVs), a viral vector for gene therapy.	Batch	Diatomaceous earth filtration	A robust and efficient alluvial filtration method for the clarification of adeno‐associated viruses from crude cell lysates.^26^

*Note*: ATF, alternating tangential flow filtration; TFDF, tangential flow depth filtration; TFF, tangential flow filtration.

As can be seen from Table 1, TFF, tangential flow depth filtration (TFDF) and alternating tangential flow filtration (ATF) appear particularly attractive for virus harvesting. In the case of virus harvesting, for both batch and perfusion operations, a cell retention device is required while the product virus particles must be recovered at high yield. The cell retention device may be classified by its method of action such as filtration, sedimentation, ultrasonic fixation and dielectrophoretic exclusion.^27^ Hollow fiber based cell retention devices are of great interest in the production of virus particles.^8^, ^9^, ^10^, ^12^, ^13^, ^15^

Virus harvesting using hollow fibers is challenging at high cell densities as is the case in the production of protein‐based therapeutics. Further, unlike protein‐based therapeutics, virus infection leads to cell apoptosis, which leads to cell lysis. Often, if a significant number of virus particles are intracellular, cell lysis is needed during virus harvesting in order to maximize the yield of virus particles. This increases the amount of impurities that can foul the membrane. Nikolay et al.^7^ provide guidelines for selecting appropriate hollow fibers.

One way to try to improve throughput is to use large pore size (larger than 0.45 μm) membranes. This can lead to the need for an additional clarification step due to the presence of unwanted cell debris in the cases of protein‐based therapeutics.^2^, ^28^ In the case of virus harvesting, studies have indicated that fouling of large pore size membranes can also lead to significant product rejection.^7^

Other recent modifications to TFF include TFDF and ATF. In TFDF, thick‐walled symmetric tubular fibers are used. These fibers contain tortuous flow paths, 2–4 μm in diameter, through the membrane. The wall thickness of the tubular membranes is around 5 mm compared to hollow fibers, which are around 0.075–0.2 mm.^11^ In effect, these tubular membranes operate like a depth filter while the feed flows tangential to the membrane surface. Recent studies suggest that TFDF does enable higher capacities than traditional hollow fibers.

ATF involves modification of the operation of traditional TFF. Unlike TFF, the feed is pumped through the module in both directions by a diaphragm pump. Initially, feed is withdrawn from the bioreactor and then it is pumped back into the bioreactor. As the feed travels through the hollow fibers in both directions, permeate is withdrawn. Flow reversal plus the generation of Starling recirculation flow in the module helps remove deposited particulate matter from the membrane surface.^29^, ^30^ While ATF leads to reduced fouling and higher productivity, it may cause an increase in shear stress on the cells, which can lead to cell damage and reduced flux.^7^

**FIGURE 1:**
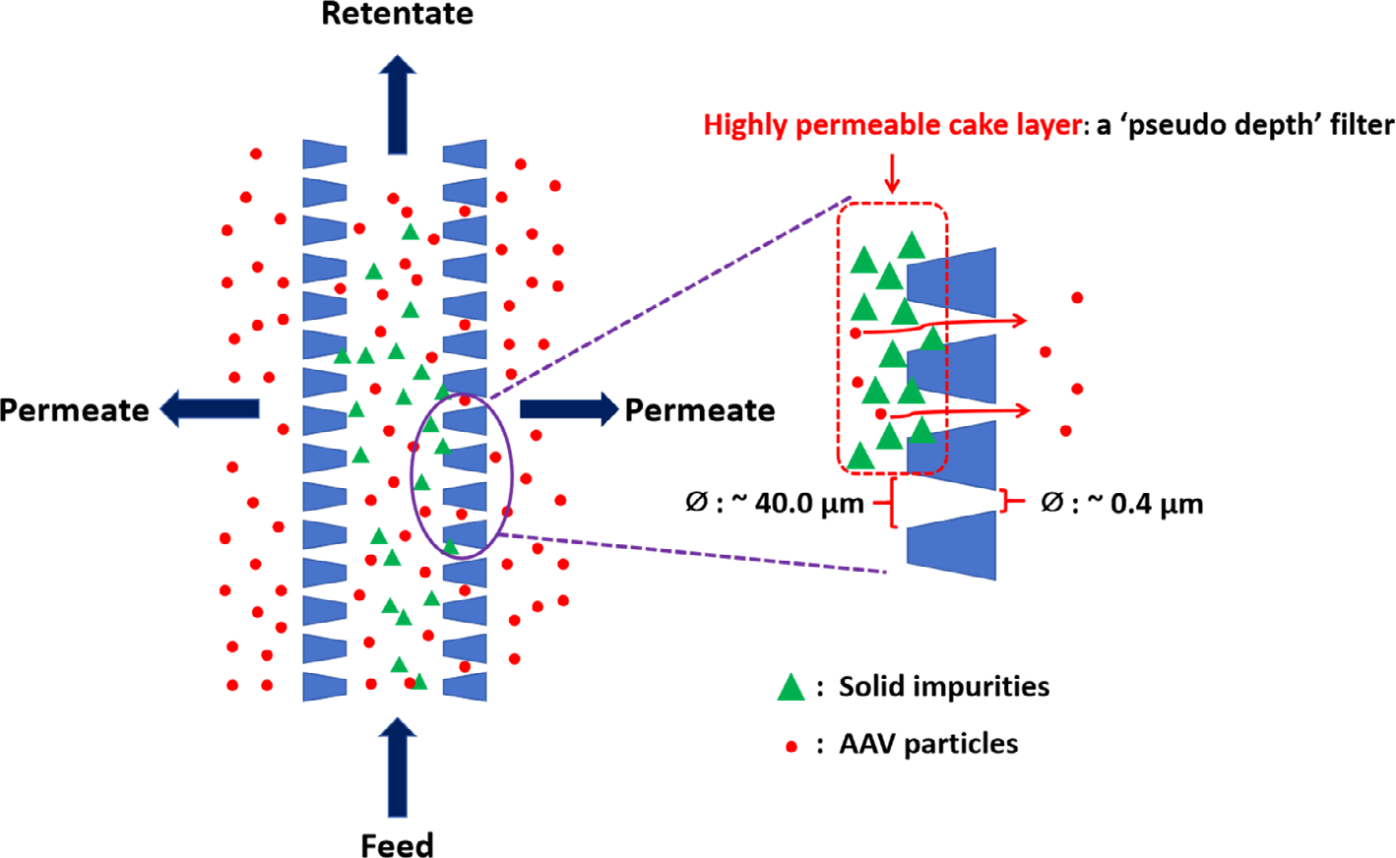
Schematic representation of the mechanism of clarification of the BioOptimal™ MF‐SL microfilter.

In our previous studies, we have shown that reverse asymmetric hollow fiber membranes (BioOptimal™ MF‐SL, Asahi Kasei Bioprocess, Glenview IL) may be used in TFF for clarification of yeast and CHO cell feed streams.^31^, ^32^ In this configuration, the more open support structure of the asymmetric hollow fiber membrane faces the feed stream. The barrier layer faces the permeate. Particulate matter is trapped by the open pore structure of the fiber lumen. The open support structure stabilizes a highly permeable cake layer in the membrane pores. The stabilized cake layer acts as a “dynamic” membrane or filter aid. These reverse asymmetric membranes act as a “pseudo depth” filter operated in TFF mode. Figure 1 is a schematic representation of the clarification mechanism of the BioOptimal™ MF‐SL microfilter. Unlike TFDF, the hollow fibers are asymmetric. The pore size in the fiber lumen is around 40 μm while it is 0.4 μm on the outer permeate side. The inside diameter of these hollow fibers is 1.4 mm, the outside diameter 2.3 mm, i.e., thickness is 0.45 mm. Thus, the membrane thickness is significantly less than the fibers used for TFDF, though a little thicker than traditionally hollow fibers used for TFF. While these hollow fiber membranes act as a depth filter which stabilizes a deposited cake layer, they also reject particulate matter based on a much tighter barrier layer. The idea of using the more open support structure to stabilize a highly permeable cake layer was proposed in the 1990s.^33^ Here, we extend our earlier work on clarification of yeast and CHO cell feed streams to clarification of cell lysates. We focus on the recovery of adeno‐associated virus (AAV) serotype 2 particles.

Adeno associated virus (AAV) is a leading candidate for gene therapy applications. AAV is a helper‐dependent mammalian parvovirus (i.e., it is replication defective). Co‐infection of the host cell by adenovirus, herpesvirus, or baculovirus is required for replication.^34^ The AAV serotype 2 is poorly released into the cell supernatant. Therefore, a lysis step is required for effective product recovery.^35^ This leads to a significant increase in particulate matter, which can lead to membrane fouling. Batch clarification of human embryonic kidney (HEK 293) cell lysate is conducted here in order to harvest AAV particles.

BioOptimal™ MF‐SL hollow fiber microfilters were used for AAV harvesting. Controlling the transmembrane pressure (TMP) is essential in order to ensure the stabilized cake layer remains highly permeable.^33^ Initial experiments were conducted in total recycle mode with and without transfection of the HEK cells. The effect of virus particles on clarification as well as the importance of determining the critical flux is determined. The critical flux is defined as the maximum stable flux.^36^ It depends on the wall shear rate (feed flow rate through the fibers) and the amount of particulate matter present. The amount of particulate matter was qualitatively determined by measuring turbidity. Diafiltration was conducted in order to maximize AAV recovery. Host cell protein (HCP) and free DNA concentrations in the various feed and permeate streams were determined. Virus titer was determined by qPCR.

## 2 MATERIALS

Gibco® viral vector HEK medium, GlutaMAX™ supplement, AAV‐MAX Transfection Kit, AAV‐MAX control plasmids kit, AAV‐MAX lysis buffer, Picogreen assay kit for DNA measurement, and Pluronic F68 were purchased from Thermo Fisher Scientific (Waltham, MA, USA). The 2100 Q turbidity meter was purchased from HACH (Loveland, CO, USA). PBS tablets, phosphoric acid (85% ACS grade) and 95% ethanol were purchased from VWR (Radnor, PA, USA). BioOptimal™ MF‐SL microfilters (50 cm^2^, number of hollow fibers: 5, length of hollow fibers: 230 mm, inside diameter of hollow fibers: 1.4 mm) were provided by Asahi Kasei Bioprocess (Glenview, IL, USA). Coomassie Brilliant Blue G‐250 was purchased from MilliporeSigma (Burlington, MA, USA). DNase and DNase buffer (10×) were purchased from Promega (Madison, WI, USA); nuclease‐free deionized (DI) water was purchased from OMEGA BIO‐TEK (Norcross, GA, USA); iTaq™ universal SYBR green supermix and PCR tubes/caps were purchased from Bio‐Rad (Hercules, CA, USA); and forward and reverse primers were purchased from Integrated DNA Technologies (Coralville, IA, USA). The AAV serotype 2 genome titers were quantified by a Bio‐Rad CFX Connect™ Real‐Time System (Hercules). The AAV serotype 2 plasmids (6208 bp, 2 × 10^9^ molecules μL^−1^, cat# 59462‐AAV2) were purchased from Addgene (Watertown, MA, USA).

## 3 METHODS

### 3.1 HEK 293 cell culture and AAV production

HEK 293 cells were cultivated in suspension using a complete growth medium formulated by supplementing Gibco® Viral Vector HEK Medium containing 2% (v/v) GlutaMAX™ supplement. The medium was warmed to 37°C before inoculation. The initial cell seeding density was set at 0.3 × 10^6^ cells mL^−1^. The culture was maintained in a CO₂ shaking incubator at 37°C with a humidified atmosphere (82% relative humidity) and continuous shaking at 250 rpm. Cell growth was monitored every 24 hours to determine the optimal time for AAV transfection. For TFF experiments that did not include AAV particles, the cells were not transferred.

When the cell density reached 5.0–6.0 × 10^6^ cells mL^−1^, the culture was diluted with fresh medium to adjust the final cell density to 3.0 × 10^6^ cells mL^−1^ for AAV capsid production. AAV plasmids were transfected into the HEK 293 cells to initiate AAV capsid production. The culture was incubated for 72 hours to allow sufficient time for AAV capsid production within the HEK 293 cells. Subsequently, 10% (v/v) AAV‐MAX lysis buffer was added to the cell medium and incubated for 4 h to lyse the cells and release the produced AAV capsids into the medium.

### 3.2 TFF

**FIGURE 2:**
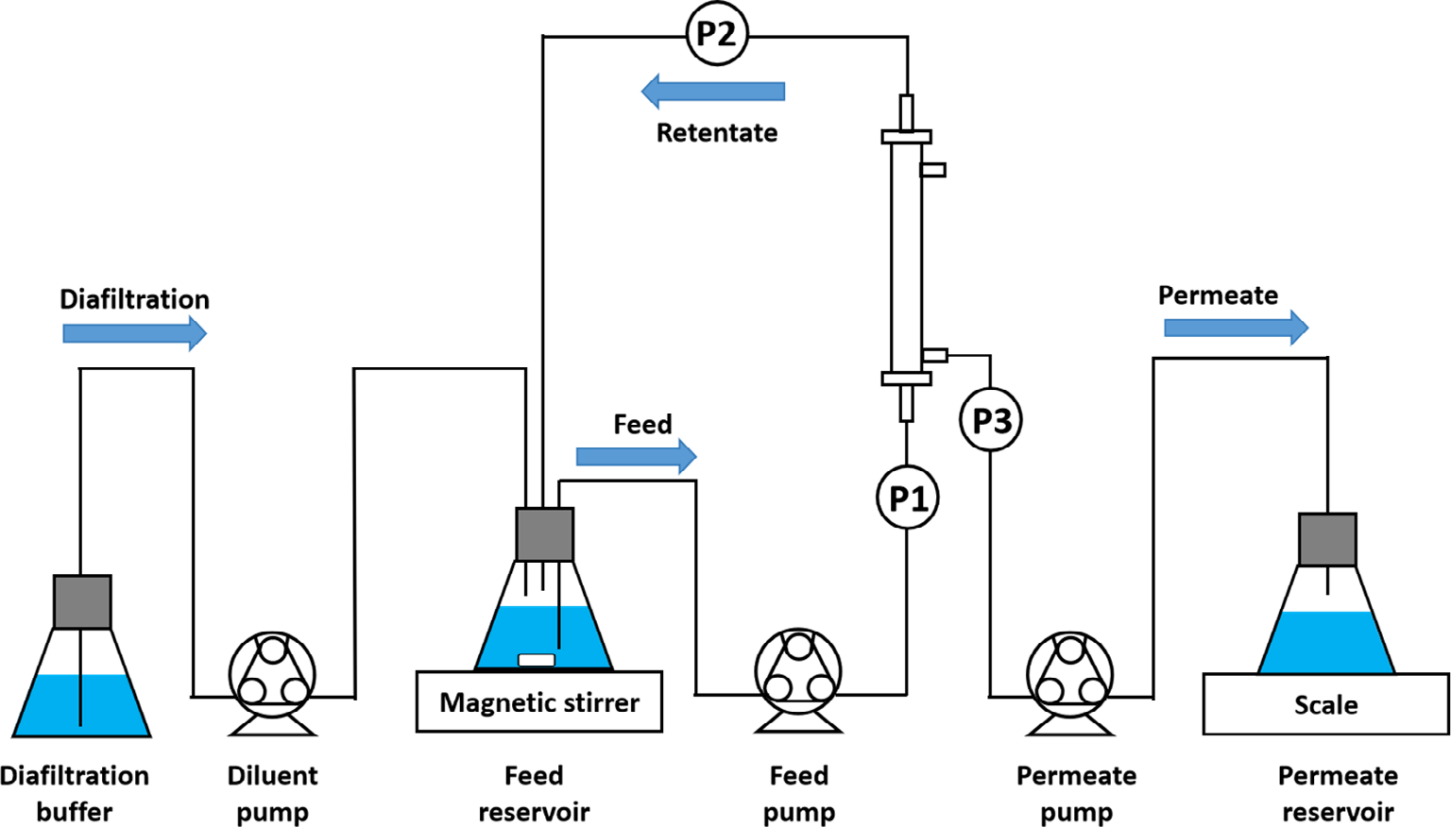
Diagram of the TFF system. P1, P2, and P3 refer to pressure transducers for the feed, retentate, and permeate lines, respectively.

The TFF system was set up as shown in Figure 2. Masterflex (Gelsenkirchen, Germany) size 16 silicone tubing was employed for all component connections. Three peristaltic pumps (Cole Parmer Masterflex L/S, Vernon Hills, IL, USA) were used: feed, permeate, and diluent. A data acquisition software (PressureMAT, PendoTECH, Plainsboro, NJ, USA) recorded feed, retentate, and permeate pressures. These measurements were then used to calculate the TMP and permeate flux according to Equations 1 and 2.

(1) $$\mathrm{TMP}=\frac{P_{1}+P_{2}}{2}-P_{3}$$

where *P*_1_, *P*_2_, and *P*_3_ are the feed, retentate, and permeate pressures, respectively.

(2) $$\text{Flux}=\frac{\mathrm{Wx}60}{\mathrm{\rho x}1000\mathrm{xA}}$$

where *W* is the recorded permeate weight increase in 1 min, *ρ* is the density of the permeate (assumed to be 1.0 g mL^−1^), and *A* is the membrane surface area, which was 0.005 m^2^.

### 3.3 Critical flux determination total recycle mode

Critical flux was determined in total recycle mode. The feed solution consisted of HEK cell lysate with and without transfection. A 250 mL HEK cell culture medium was lysed upon reaching a cell density of 5.0–6.0 × 10^6^ cells mL^−1^. Each experiment started immediately after cell lysis was complete. Flux stepping experiments were conducted. Feed flow rates of 8, 32, 80, 160, and 240 mL min^−1^ corresponding to wall shear rates of 100, 400, 1000, 2000, and 3000 s^−1^ were tested. Initially, the feed pump was started, and the feed was recirculated through the module for a few minutes, after which the permeate pump was started. For wall shear rates of 100 and 400 s^−1^, the initial permeate flux was set at 1 Lm^−2^ h^−1^. Permeate was collected for about 90–100 min. About 8 mL was removed for turbidity analysis. After turbidity analysis, all the permeate was returned to the feed reservoir.

For higher wall shear rates, the initial permeate flux was set at 3 Lm^−2^ h^−1^. The permeate flux was increased in increments of 2 Lm^−2^ h^−1^ for all wall shear rates. Permeate was collected for 30 min. Again, about 8 mL of permeate was used to measure turbidity. For non‐transfected cells, the entire permeate was returned to the feed reservoir. For transfected cells (TFF conducted at 2000 s^−1^ only) 1 mL of permeate was removed for HCP, DNA, and qPCR analysis.

### 3.4 TFF and Diafiltration for AAV recovery

The experiment utilized 250 mL of AAV‐transfected HEK cell medium as the feed solution. The feed flow rate of 160 mL min^−1^, corresponding to a shear rate of 2000 s^−1^. The start‐up procedure was the same as for total recycle mode where the feed pump was started before the permeate pump. The permeate pump operated at a flow rate of 1.25 mL min^−1^ (15 Lm^−2^ h^−1^). Permeate was collected in 50 mL fractions throughout the process for turbidity, HCP, DNA, and qPCR analysis. The TMP was also monitored. The diafiltration started once the TMP reached 3.5 kPa (0.5 psi). This occurred after about 150 min of operation. The diluent was pumped, here PBS buffer, at the same flow rate as the permeate pump. The total volume of PBS used was three times the residual feed volume (3 diavolumes).

### 3.5 Turbidity

Turbidity measurements were performed using a Hach 2100Q turbidimeter. Ten milliliters of each sample was carefully transferred into a sample cell. The percentage turbidity reduction was calculated according to Equation (3).

(3) $$\text{Turbidity reduction}=\left(1-\frac{\text{Pemeate turbidity before critical flux}}{\text{Feed turbidity}}\right)\times 100\%$$

### 3.6 HCP and DNA analysis

HCP was determined using the Bradford assay. The Bradford reagent was prepared by dissolving 50 mg of Coomassie Brilliant Blue G‐250 in 25 mL of 95% ethanol. Then, 50 mL of 85% phosphoric acid and 425 mL of deionized water were added. Briefly, 75 μL of the sample and 125 μL of the Bradford reagent were mixed well in a cuvette and then incubated in the dark for 10 min. Absorbance at 595 nm was subsequently measured using a microplate reader. The absorbance was converted into protein concentration using a standard curve of known concentration of bovine serum albumin (BSA) from 0.1 to 1 g L^−1^ using the same method.

DNA concentration was quantified using the PicoGreen assay. A working solution of PicoGreen® dye was prepared by diluting the concentrated stock 1:199 with 10 mM Tris–HCl buffer (pH 7.5) containing 1 mM EDTA. In a 96‐well plate, 100 μL of the sample was mixed thoroughly with 100 μL of the PicoGreen working solution. The plate was then incubated in the dark at room temperature for 5 min. The fluorescence intensity of the PicoGreen complex was measured using a microplate reader at an excitation wavelength of 502 nm and an emission wavelength of 522 nm. A standard curve was constructed by plotting the fluorescence intensity of known DNA standards versus their corresponding concentrations. The concentration of unknown DNA in the samples was then calculated by interpolation from the standard curve.

### 3.7 Determination AAV titer by qPCR

DNase treatment solution was prepared for each sample by mixing DNase, DNase buffer, 0.1% Pluronic F68, and nuclease‐free DI water in the ratio of 5:5:5:30 μL. The solution was dispensed into a PCR tube, total volume 45 μL. Five microliters of the sample was added into the tube (final dilution of DNase buffer was tenfold) and mixed well by pipetting the solution up and down 20 times. The tube was then capped and loaded into the tube holder of the CFX Connect qPCR instrument (Hercules). DNase treatment was conducted at 37°C for 30 min.

The DNase‐treated sample was diluted 100 times with nuclease‐free DI water. The amplification solution was prepared by mixing the SYBR green supermix, nuclease‐free DI water, and forward and reverse primers in a ratio of 10:4.7:0.15:0.15 μL (total volume 15 μL). Then, the mixture was dispensed into a PCR tube. Five microliters of the diluted sample was added to the PCR tube and well mixed by pipetting up and down 20 times. After capping, the tube was loaded into the tube holder.

The amplification protocol involved the following steps: initiation/pre‐denaturation at 98°C for 3 min, followed by up to 39 cycles of the amplification at 98°C for 15 s and 58°C for 30 s, followed by 65°C for 3 s and 95°C for 30 s. The viral titer was then calculated from a standard curve generated by plotting the threshold cycle values, *C*_t_, against the logarithm of the initial concentration of standard AAV serotype 2 genome ranging from 3.0‐ to 8.0‐log μL^−1^. Ct is defined as the number of cycles required to produce a constant fluorescence emission. This standard curve was prepared using AAV serotype 2 plasmids (6208 bp, 2 × 10^9^ molecules μL^−1^, cat# 59462‐AAV2) purchased from Addgene (Watertown, MA, USA) following the amplification protocol as above.

## 4 RESULTS

**FIGURE 3:**
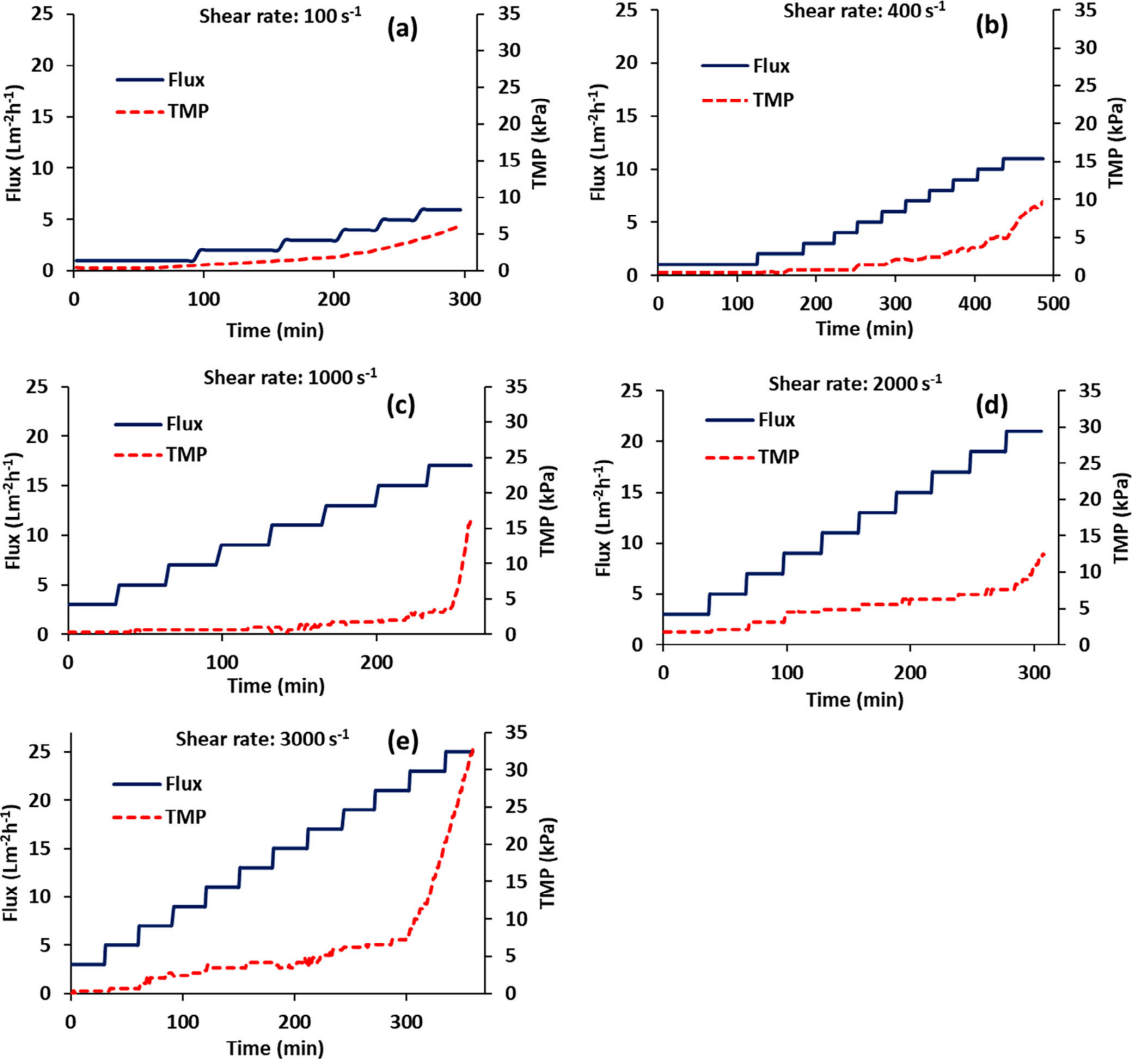
Variation of permeate flux and TMP with time. (a–e) Wall shear rates of 100, 400, 1000, 2000, 3000 s^−1^, respectively. The cells were not transfected.

**FIGURE 4:**
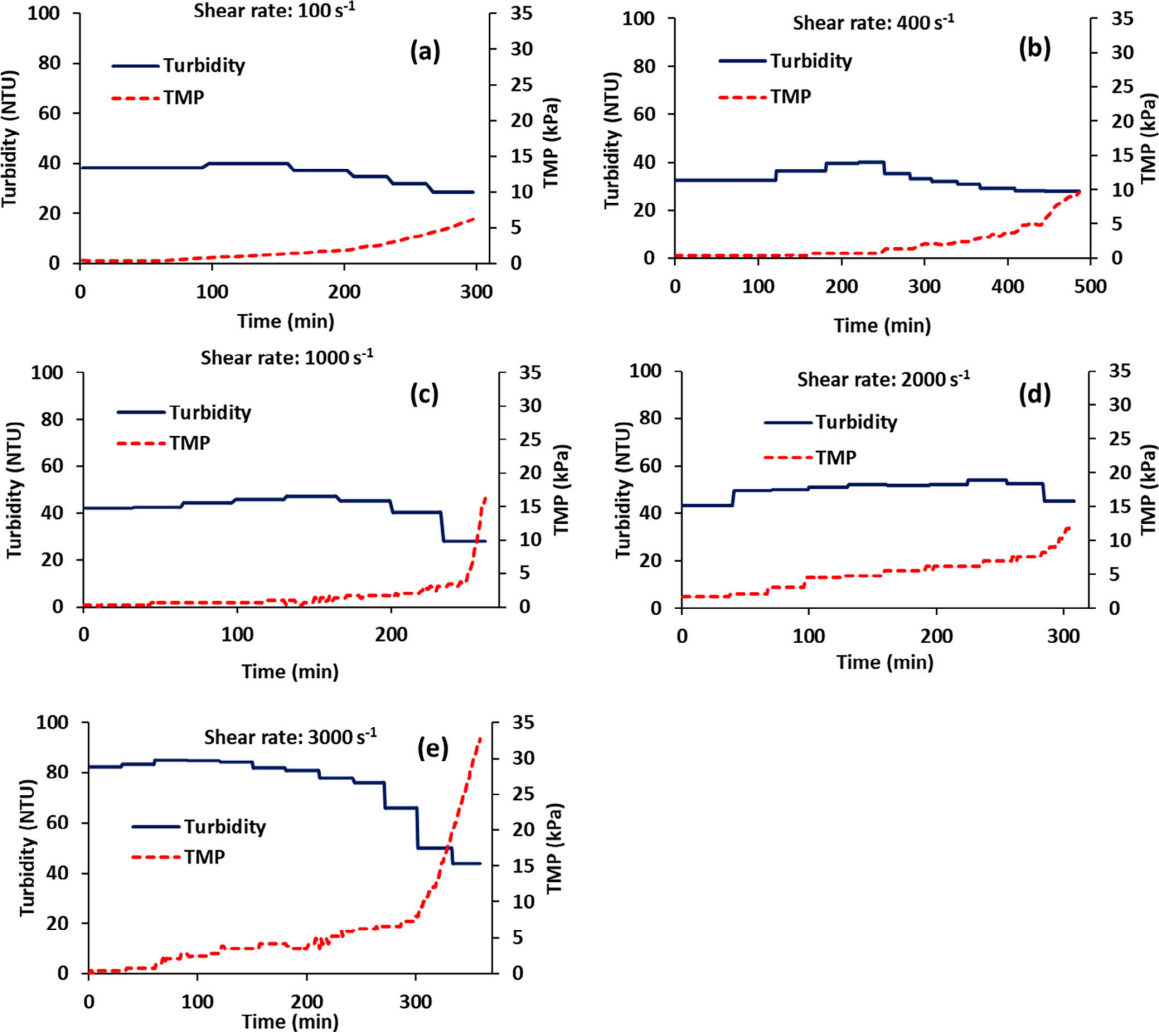
Variation of permeate turbidity and TMP with time. (a–e) Wall shear rates of 100, 400, 1000, 2000, 3000 s^−1^. The cells were not transfected.

Figures 3 and 4 give results for flux stepping experiments for non‐transfected HEK cells conducted in total recycle mode. Figure 3 gives the variation of permeate flux and TMP with time while Figure 4 gives the variation of permeate turbidity and TMP with time. Figures 3a–e and 4a–e represent wall shear rates of 100, 400, 1000, 2000, 3000 s^−1^. Figure 3a–e indicates that at permeate flux values of 5, 10, 17, 21, 23 Lm^−2^ h^−1^, respectively, the TMP begins to increase rapidly. This indicates that the critical flux has been exceeded as the flux is no longer stable.

Figure 4 indicates that the initial turbidity of the permeate is similar at all wall shear rates except for 3000 s^−1^. Table 2 gives the initial cell density and turbidity of the feed after cell lysis. As can be seen, the cell densities prior to cell lysis are relatively uniform for all wall shear rates investigated. They are in the range of 5.0–5.5 × 10^6^ cells mL^−1^. The initial feed turbidity after cell lysis is also relatively uniform, being between 240 and 280 NTU. For non‐transfected cells, the higher initial turbidity of the permeate for a wall shear rate of 3000 s^−1^ is probably due to higher shear resulting from the higher feed flow rate through the peristaltic pump, leading to the production of smaller cellular fragments. Consequently, higher wall shear rates, above 2000 s^−1^, were not investigated for AAV recovery.

**TABLE 2:** HEK cell density and cell lysate turbidity.

Shear rate (s^−1^)	Cell density (10^6^ mL^−1^)	Cell lysate turbidity (NTU)
100	5.3	265
400	5.5	280
1000	5.3	239
2000^a^	5.6	620
2000^b^	5.1	480
2000	5.0	240
3000	5.5	260

^a^ AAV‐transfected HEK cells for flux stepping experiment (Figures 7 and 8).

^b^ AAV‐transfected HEK cells for AAV harvesting (Figures 9 and 10).

For all wall shear rates, the permeate turbidity increases and then decreases, especially as membrane fouling occurs and the TMP begins to rise at permeate fluxes above the critical flux. Once the membrane pores are plugged and the stabilized cake is no longer highly permeable, it is expected that the rejection properties of the membrane will change, leading to greater rejection of smaller particulate matter and a decrease in permeate turbidity.

**FIGURE 5:**
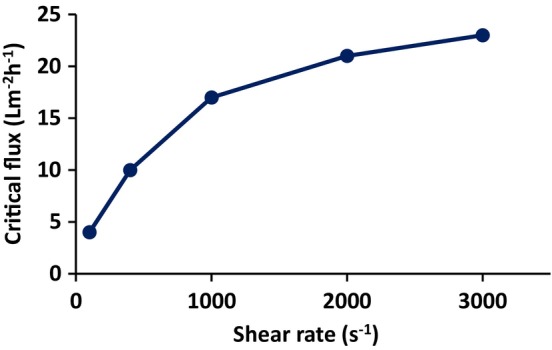
Variation of critical flux with wall shear rate.

The critical flux depends on wall shear rate. Figure 5 gives the variation of critical flux with wall shear rate. As can be seen the critical flux increases with increasing wall shear rate. Figure 5 indicates an appropriate critical flux for a given wall shear rate.

**FIGURE 6:**
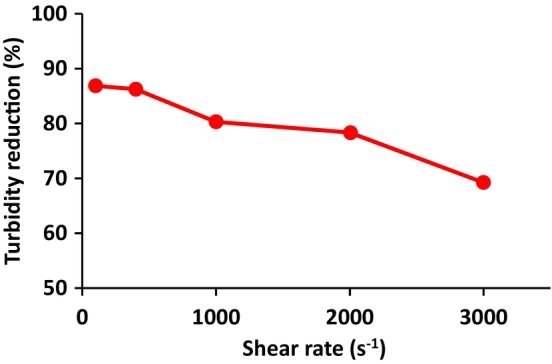
Variation of percent turbidity reduction with wall shear rate as given by Equation (3).

Figure 6 gives the variation of permeate turbidity reduction in the bulk permeate, as defined by Equation (3), as a function of wall shear rate. Turbidity reduction of the permeate relative to the feed rather than the turbidity of the permeate is used, as the actual turbidity of the feed and permeate vary for different experiments. This is not unexpected. The reduction in turbidity decreases with increasing wall shear rate.

**FIGURE 7:**
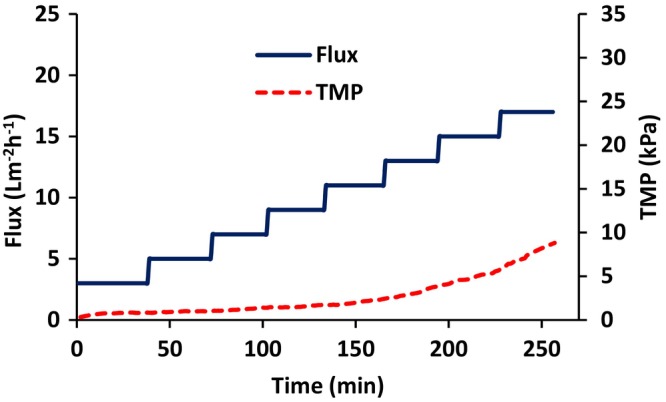
Variation of permeate flux and TMP with time for transfected HEK cells at a wall shear rate of 2000 s^−1^.

**FIGURE 8:**
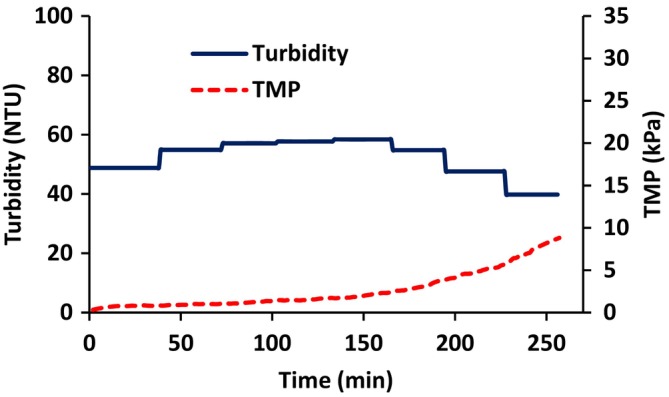
Variation of permeate turbidity and TMP with time for transfected HEK cells at a wall shear rate of 2000 s^−1^.

Figures 7 and 8 give the variation of permeate flux and TMP with time and turbidity and TMP with time, respectively, for transfected HEK cells that produced AAV particles. The flux stepping experiment was run under total recycle. A wall shear rate of 2000 s^−1^ was selected. Figures 5 and 6 suggest that at higher wall shear rates, the turbidity increases due to more rapid pumping of the feed through the BioOptimal™ MF‐SL microfilter. Furthermore, 2000 s^−1^ is a common wall shear rate in industrial practice.

Comparing Figure 7 with Figure 3d it can be seen that in the presence of virus particles, the critical flux is lower. It is about 15 Lm^−2^ h^−1^ for transfected HEK cells, whereas it was 21 Lm^−2^ h^−1^ for non‐transfected cells. Comparing the initial feed turbidity for non‐transfected cells (240 NTU) and transfected cells (620 NTU, see Table 2) after cell lysis it can be seen that the feed turbidity is higher for transfected cells. This agrees with the lower critical flux. The turbidity reduction for non‐transfected cells was 80% (Figure 6) versus 90% for transfected cells (calculated by Equation 3).

Based on the results of flux stepping experiments for transfected and non‐transfected HEK cells, an experiment was conducted in order to recover AAV particles. The critical flux was taken as 15 Lm^−2^ h^−1^ based on Figure 7. Thus, the permeate flux was set at 15 Lm^−2^ h^−1^. Permeate was collected in 50 mL (approximately 42 min) fractions. Each fraction was analyzed for turbidity, HCP, DNA, and virus titer. The TMP was monitored. When the TMP increased to 3.5 kPa, diafiltration commenced using 3 diavolumes of diluent. Diafiltration commenced during the collection of permeate fraction 4. When diafiltration started, 64 mL of feed was left in the feed reservoir. Consequently, the total volume of diluent added was 192 mL.

**FIGURE 9:**
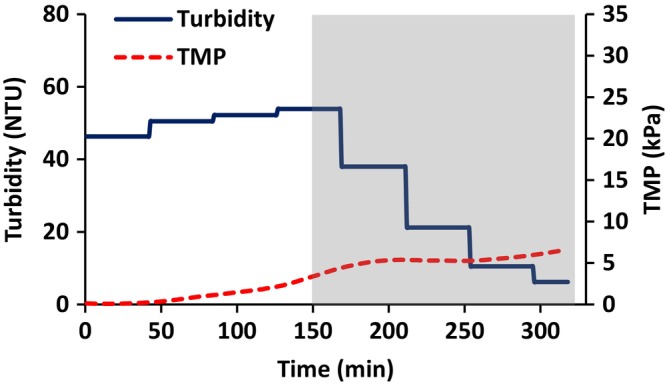
Variation of permeate turbidity and TMP with time for AAV containing feed stream. Diafiltration started at 150 min.

Figure 9 gives the variation of permeate turbidity and TMP with time. As can be seen, the turbidity of the permeate initially increases. When diafiltration commences at 150 min, it begins to decrease. The discrete steps in the variation of permeate turbidity with time are a consequence of the fact that the permeate was analyzed in 50 mL fractions. As the feed solution is concentrated, the TMP increases. At the commencement of diafiltration, the TMP stabilizes but begins to increase again at the end of the run.

**FIGURE 10:**
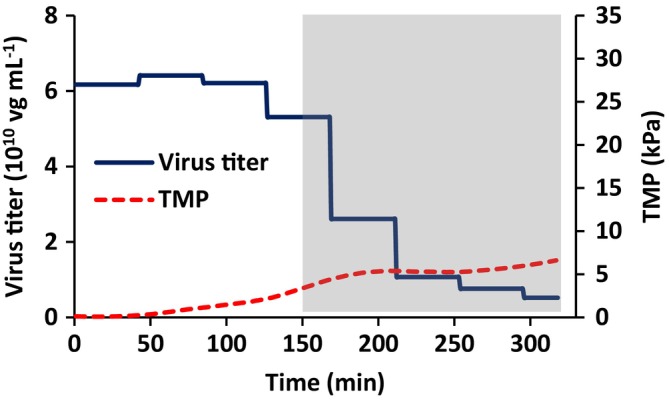
Variation of AAV titer and TMP with time. Diafiltration commenced at 150 min. Initial feed titer was 6.2 × 10^10^ μg mL^−1^.

Figure 10 gives the variation of virus titer and TMP with time. The discrete steps in the variation of virus titer with time are a consequence of the fact that the permeate was analyzed in 50 mL fractions. The virus titer in the permeate is essentially constant during concentration. The actual AAV titer in each permeate fraction as well as the percentage of total AAV particles present in the feed is given in Table 3. The AAV titer in the permeate decreases during diafiltration, as is expected, as the feed is being diluted. Importantly, it appears there is little rejection of AAV particles. Total AAV recovery is 94%.

**TABLE 3:** AAV titer in each permeate fraction as well as percentage of total AAV in feed. Initial feed titer was 6.2 × 10^10^ μg mL^−1^.

Sample	Volume (mL)	AAV titer (10^10^ μg mL^−1^)	% of total particle number to feed
Feed	250.0	6.2	100.0
Permeate 1	50.0	6.3	20.4
Permeate 2	50.0	6.4	20.7
Permeate 3	50.0	6.2	20.1
Permeate 4	50.0	5.3	17.2
Permeate 5	50.0	2.6	8.4
Permeate 6	50.0	1.1	3.6
Permeate 7	50.0	0.8	2.6
Permeate 8	35.0	0.5	1.1
Total AAV recovery in bulk permeate			94.1
Retentate	65.0	0.7	2.5

Table 4 gives the HCP and DNA concentration in each permeate fraction and final retentate pool. As was the case for AAV particles, for HCP the concentration in the feed and the first two permeate fractions is similar suggesting little rejection by the membrane. The concentration decreases during diafiltration due to dilution of the feed. In the case of DNA, the higher concentration in the feed compared to permeate fraction 1, is probably due to rejected cells and cellular fragment associated DNA. During diafiltration the DNA concentration in the permeate decreases as is expected due to dilution of the feed.

**TABLE 4:** HCP and DNA in each permeate fraction.

Sample	Volume (mL)	HCP (mg mL^−1^)	DNA (μg mL^−1^)
Feed	250.0	1.78	8.18
Permeate 1	50.0	1.70	4.52
Permeate 2	50.0	1.69	5.37
Permeate 3	50.0	1.44	5.19
Permeate 4	50.0	1.31	5.43
Permeate 5	50.0	0.68	3.60
Permeate 6	50.0	0.36	1.90
Permeate 7	50.0	0.25	1.10
Permeate 8	35.0	0.21	1.03
Retentate	65.0	0.27	2.22

## 5 DISCUSSION

Arora and Davis^37^ show that the formation of a thin, highly permeable secondary cake layer on a membrane surface during normal flow filtration can lead to higher fluxes than in the absence of the cake layer. The secondary cake layer acts as a filter aid, removing foulants that would otherwise adsorb on the membrane, leading to an increase in product rejection and a drop in permeate flux. However, ensuring the layer of deposited cellular material is thin and highly permeable is essential.

Guerra et al.^33^ show that by using an inverse asymmetric membrane, as is the case in this study, the membrane support structure stabilizes a thin and highly permeable secondary cake layer. Here we extend this concept originally demonstrated in our earlier work for clarification of yeast cells and Chinese Hamster Ovary (CHO) cells feed streams^31^, ^32^ to cell lysates. While the support structure of the asymmetric membrane stabilizes a thin cake layer, the TMP must be chosen to prevent compaction of the stabilized cake layer.

The concept of critical flux, the flux below which no fouling occurs, was first described in 1995 by Field et al.^38^ Since that time, numerous theoretical and experimental studies have been conducted to better understand the meaning of “no fouling” and the important variables that affect the critical flux. Bacchin et al.^39^ recently reviewed theoretical development and experimental evidence for the existence of a critical flux. While from a theoretical perspective, strong and weak forms of the critical flux have been defined,^40^ from a practical perspective, interest in the concept of sustainable flux has grown significantly.^36^ The definition of sustainable flux is much vaguer. Howell^41^ indicates that the deposition of a thin cake layer of rejected yeast cells can lead to an increased sustainable flux as the yeast cells act as a filter aid.

From an industrial perspective, the sustainable flux represents an acceptable rate of fouling between the cleaning cycles of batch operations. The sustainable flux will depend on economic considerations. Clearly, for perfusion operations, where TFF must operate for much longer times, the sustainable flux will be different from batch operations where shorter run times and higher processing rates are required. Unlike the critical flux, which is strongly based on the theory of particle‐particle and particle‐membrane interactions, the sustainable flux is influenced by hydrodynamic conditions, feed conditions, and the required process time.

Compaction of the stabilized secondary membrane for reverse asymmetric membranes will lead to rapid fouling. Here we estimate the critical flux by conducting flux stepping experiments. We define the critical flux as the maximum flux for which the TMP does not rapidly increase during 30 min of operation. Determining an appropriate sustainable flux is particularly important for reverse asymmetric membranes. In the case of TFDF, since the entire membrane acts as a depth filter, it is perhaps less critical, as the aim is not to form a thin, highly permeable secondary membrane consisting of rejected particulate matter that acts as a filter aid.

Permeate turbidity has been used as a quick method to determine the effectiveness of particulate matter removal. As can be seen, the permeate turbidity increases with increasing flux. This is probably due to increasing convective flow through the membrane that forces smaller particulate matter through the 0.4 μm barrier layer. Nevertheless, the turbidity is generally around 40–50 NTU with the exception of a wall shear rate of 3000 s^−1^. The decrease in permeate turbidity above the critical flux agrees with the fact that above the critical flux, compression of the stabilized cake layer will lead to an increase in TMP for a given flux as well as a change in rejection properties of the membrane.

Deposition of particulate matter on the membrane surface depends on a balance between convection toward the membrane and back migration away from the membrane surface due to mechanisms such as shear enhanced diffusion and inertial lift forces.^42^ The rate of back migration depends on particle size. Figures 7 and 8 indicate similar trends to Figures 3 and 4 for transfected and non‐transfected HEK cells, respectively. The main difference is that the initial feed turbidity is higher for transfected HEK cells (Table 2). In the case of transfected cells, in addition to the cell lysis that is conducted prior to virus harvesting, virus infection frequently leads to cell apoptosis, which results in cell lysis. This could explain the higher initial feed turbidity. The higher feed turbidity results in a higher concentration of particulate matter during TFF, which leads to critical flux around 15 Lm^−2^ h,^−1^ which is lower than 21 Lm^−2^ h^−1^ observed for non‐transfected cells at a wall shear rate of 2000 s^−1^. This also leads to a slightly higher permeate turbidity. The decrease in percent turbidity reduction in the permeate at higher wall shear rates (see Figure 6 for non‐transfected cells) is probably due to the increased breakdown of cellular debris into smaller particles at higher wall shear rates as well as differences in the stabilized secondary cake layer at higher wall shear rates.

It can be seen that the reduction in permeate turbidity relative to the feed (Equation 3) at a wall shear rate of 2000 s^−1^ for non‐transfected HEK cells is about 80% (Figure 6). For transfected HEK cells during virus harvesting, using Equation (3) to calculate the percent turbidity reduction in the bulk permeate gives 90%. The higher value may be due to the fact that the permeate is diluted during diafiltration. In the experiments conducted here, as the operation is maintained below the critical flux, it is not possible to determine the reduction in permeate turbidity before the onset of rapid flux decline, as is the case for flux stepping experiments. However, by using inline turbidity measurement, it will be possible to better control the process.

The results for AAV harvesting (Figures 9 and 10) provide insights into the operation of reverse asymmetric membranes for bioreactor clarification. The initial flux is set at a “sustainable flux” which is below the critical flux. As the contents of the feed reservoir are concentrated, the TMP increases. As the TMP increases, a slow increase in turbidity of the permeate is observed. Importantly, Figure 10 and Table 3 indicate that feed and virus titers before diafiltration are within 0.1 log unit, indicating no AAV rejection. Here we set the maximum TMP at 3.5 kPa or 0.5 psi, after which diafiltration commenced in order to maximize AAV recovery. It is essential that the maximum TMP does not lead to permanent deformation of the stabilized cake layer and hence a continual increase in TMP and rejection of AAV. Here the TMP remains stable, increasing slightly at the end of the run. In the experiment conducted here, the total throughput was 756 Lm^−2^ consisting of 186 mL of feed solution during concentration and an additional 192 mL during diafiltration. The run was terminated as three diavolumes had been processed and further concentration of the feed was not possible due to the required hold‐up volume of the system. However, Figures 9 and 10 suggest that higher membrane throughputs are possible and should be investigated when developing a commercial process.

From a practical perspective, this study indicates that initial flux stepping experiments are essential in order to estimate the critical flux. TFF should always be operated below the critical flux. Operating close to the critical flux will minimize the batch processing time. On the other hand, for perfusion operations, ensuring operation at a sustainable flux for long periods of time is essential.

Diafiltration is essential to maximize AAV recovery. Diafiltration must be commenced before the TMP is high enough to cause permanent compression and deformation of the stabilized cake layer. From a practical perspective, bioreactor clarification could commence at a flux that leads to 50% of the TMP at the critical flux. The TMP (at constant flux) will increase as the contents of the bioreactor are concentrated. When the TMP reaches 80% of the value observed at the critical flux, diafiltration could commence. By carefully monitoring changes in TMP and turbidity online, the point at which diafiltration commences can be more carefully determined and controlled. Commencing diafiltration early in the process will require use of additional diluent and lead to a lower virus titers in the bulk permeate. Here we show that at a TMP of 3.5 kPa, the stabilized cake layer appears to remain permeable. The results obtained here indicate that using a reverse asymmetric membrane, operated under appropriate flux and TMP conditions, could lead to an efficient unit operation for virus harvesting. It is likely that the sustainable flux and maximum TMP for batch operation will be different for continuous operation due to the different product throughput and much longer TFF run times that are required for continuous operation.

For commercial applications, scalability is essential. BioOptimal™ MF‐SL microfilters are available in a range of sizes with membrane surface areas as high as 8 m^2^. The manufacturer indicates throughputs of up to 300 L m^2^ at high cell viabilities, for example, over 90%. In earlier studies where we investigated the clarification of CHO cells feed streams in batch mode with cell viabilities ranging from 0 to 96%, a rapid drop in flux and consequently throughput is observed at cell viabilities below 70%.^43^ By carefully controlling the TMP at around 50% of the TMP at the critical flux and commencing diafiltration at around 80% of the TMP at the critical flux, much higher throughput and around 95% recovery of AAV is expected.

## 6 CONCLUSION

The use of reverse asymmetric hollow fiber membranes for clarification of cell lysates has been studied. The results indicate the importance of carefully controlling the maximum flux and TMP. The maximum sustainable flux may be estimated by calculating the critical flux. Flux stepping experiments under total recycle provide a rapid method to estimate the critical flux. The critical flux was found to increase with increasing wall shear rate. AAV2 harvesting was conducted at a permeate flux of 15 Lm^−2^ h^−1^, the critical flux estimated from flux stepping experiments. As the feed is concentrated, the TMP increases. Figure 7 indicates that the TMP should be kept below about 3.5 kPa in order to prevent compaction of the stabilized layer of rejected particulate matter. Thus, diafiltration commenced at this TMP. Here, 3 diavolumes of diluent were used. Diafiltration is important to maximize recovery of AAV2. It is important to determine the optimal time to commence diafiltration. Commencing diafiltration early in the process increases diluent usage and increases product dilution. However, if the TMP is too high, the secondary cake layer may have a very low permeability and rejection of AAV2 is possible. Further, carefully controlling the TMP at which diafiltration commences will maximize the membrane throughout Throughputs higher than 300 Lm^−2^ are obtainable, and as high as 750 Lm^−2^ may be possible. The TMP did not increase significantly during diafiltration, and AAV2 recovery was 94%. The results indicate that reverse asymmetric membranes could be well suited for clarification of cell lysates, provided the permeate flux and TMP are carefully controlled.

## CONFLICT OF INTEREST STATEMENT

The authors declare no conflict of interest.

## Data Availability

The data that support the findings of this study are available from the corresponding author upon reasonable request.
